# Frequency of Educational Computer Use as a Longitudinal Predictor of Educational Outcome in Young People with Specific Language Impairment

**DOI:** 10.1371/journal.pone.0052194

**Published:** 2012-12-27

**Authors:** Kevin Durkin, Gina Conti-Ramsden

**Affiliations:** 1 School of Psychological Sciences and Health, University of Strathclyde, Glasgow, United Kingdom; 2 School of Psychological Sciences, The University of Manchester, Manchester, United Kingdom; Macquarie University, Australia

## Abstract

Computer use draws on linguistic abilities. Using this medium thus presents challenges for young people with Specific Language Impairment (SLI) and raises questions of whether computer-based tasks are appropriate for them. We consider theoretical arguments predicting impaired performance and negative outcomes relative to peers without SLI versus the possibility of positive gains. We examine the relationship between frequency of computer use (for leisure and educational purposes) and educational achievement; in particular examination performance at the end of compulsory education and level of educational progress two years later. Participants were 49 young people with SLI and 56 typically developing (TD) young people. At around age 17, the two groups did not differ in frequency of educational computer use or leisure computer use. There were no associations between computer use and educational outcomes in the TD group. In the SLI group, after PIQ was controlled for, educational computer use at around 17 years of age contributed substantially to the prediction of educational progress at 19 years. The findings suggest that educational uses of computers are conducive to educational progress in young people with SLI.

## Introduction

Computer use draws on linguistic abilities. To conduct a search, explore a site, prepare a document, make a purchase, download audiovisual materials, register and participate in an online community, exchange email messages, or contact one’s friends via Facebook or Skype, all entail at least minimal and often substantial amounts of language. These everyday activities require vocabulary knowledge, semantic, syntactic and pragmatic competence, literacy skills and text processing. The involvement of language in our educational, occupational and leisure uses of computers is so pervasive that we take it for granted.

Yet, for some people, language itself presents challenges, in computer use as in other aspects of their lives. One such group is people with Specific Language Impairment (SLI), a developmental condition involving difficulties understanding and/or producing language, despite having hearing and intelligence scores within the normal range [1]–[3]. Approximately 7% of children present with SLI at school entry [4]. It is one of the most common childhood impairments, yet is markedly under-represented in research into neurodevelopmental disorders [5].

While early intervention can help, many individuals diagnosed with SLI in early childhood continue to experience difficulties with language throughout childhood and adolescence, and into adulthood [6]–[8]. Even children with histories of SLI who are deemed to have “resolved” (i.e., their scores on language instruments have improved to the extent that they now fall within the typical range) can still experience language-related problems (such as reading) and other information processing difficulties in later childhood [9]–[11]. Much remains to be understood about how these individuals develop, how they cope with daily tasks, and how they can be supported to achieve optimal outcomes.

Like other young people, children and adolescents with SLI are growing up in a world in which skills in at least basic computer uses are encouraged and expected. In this study, we investigate whether home uses of computers impede or support educational progress among young people with SLI during the crucial transition period following the end of compulsory schooling.

Durkin, Conti-Ramsden, Walker and Simkin [12] review several reasons to expect that young people with linguistic impairments would be disadvantaged in the face of language-dependent modes of communication, interaction and learning. These include difficulties in the production and comprehension of written text, poorer vocabulary growth, difficulties in working memory, speed of information processing, visuo-spatial performance, auditory processing, and fine motor movements. Because uses of computer technology draw on these and related processing abilities, at least some aspects of computer work can be challenging for young people with SLI. Comparing adolescents with SLI and adolescents with typical development (TD), Durkin et al. [12] found that the SLI group scored lower on a measure of perceived ease of use of computers. Ease of use predicted frequency of use among participants with SLI but not among those with TD. In response to open-ended questions about computer applications, participants with SLI reported that they found that the information provided was too technical, involved the use of too much text, and was difficult to understand; many indicated that they found it hard to read, write and spell when using the applications.

Experiencing difficulties in using computers is associated with computer anxiety, which theory and research indicate impacts on perceived self-efficacy and in turn is likely to lead to lower usage of computers [13]–[16]. Consistent with these assumptions, Conti-Ramsden, Durkin and Walker [17] found that adolescents with SLI reported higher levels of computer anxiety than did typically developing peers.

Nevertheless, there are many reasons for young people to use computers and also some attractions to doing so. Durkin et al. [12] found that, despite facing challenges and experiencing computer anxiety, adolescents with SLI did use computers at home for both interpersonal and educational purposes, even though frequency of use was lower than for people with typical development. As with typical youth, interpersonal uses were preferred. A significantly larger proportion of adolescents with SLI did not use educational applications in a typical week (nearly one third for SLI versus only 8% for TD). Examination of those who did use educational applications also revealed differences across groups: adolescents with SLI used a number of online and offline educational applications less often than did TD youth (downloading educational materials, online libraries, the Internet to revise for exams, spreadsheets/databases and presentational software). These data indicate that a considerable proportion of adolescents with SLI experience difficulties with educational applications at home and many have little engagement. Yet, most engage with the interpersonal functions and some do persevere to use their home computers for educational purposes.

This prompts the question of whether sustained engagement with computer uses is beneficial for adolescents with SLI. The issue is particularly significant for young people with SLI because these individuals also suffer broader educational disadvantages. Their educational achievements tend to lag behind those of their peers through the school years [18]–[20]. Hence, in general, educational work is likely to be more arduous for these individuals. In this study, we examine whether two types of home computer use – leisure and educational – are associated with, and predictive of, educational progress in young people with SLI (and TD comparisons) during adolescence.

### Leisure Versus Educational Uses of Home Computers

Although increasing numbers of young people have computer and internet access at home, their preferred uses tend to be for leisure, i.e., interpersonal and entertainment rather than for educational purposes [21] [12] [22]–[24]. It is controversial whether leisure uses have negative or positive correlates and consequences. It is possible, for example, that the popular activity of playing videogames could divert children from educational activities, but it is also possible that it could promote confidence in using computers, support conceptual learning, stimulate visuo-spatial skills, and facilitate peer sociability [25]–[28] [24]. In typical youth, playing videogames has been found to be associated with a range of positive developments [29]–[31], though the direction of causality is uncertain. Similarly, it could be argued that using computers for interpersonal communication encourages the use of impoverished grammar and poor spelling and wastes time that might be spent studying; but it is also possible, and evidence confirms, that it provides an enjoyable context for the spontaneous use of writing skills [32]. Kuhlmeier and Hemker [33] found a strong relationship between home use of computers (for surfing, emailing, chatting, text processing) and Internet/computing skills among 13- to 15-year-olds. Use of ‘text language’, the distinctive form of language that has evolved among users of SMS and email, with abbreviations, slang and creative word-letter-symbol combinations, has been found to be associated with stronger literacy abilities, both in TD children [34]–[35] and adolescents with SLI [36]. Thus, there are reasons to expect that leisure uses of computers could be beneficial for young people with SLI.

With respect to educational uses, the benefits of using computers for these purposes are widely assumed but not ubiquitously demonstrated [37]–[38]. Despite the increasing scope and availability of applications in recent decades, and widespread endorsement by governments, some educators, and many parents [39] [24] [40]–[41], the use of computers for study-related activities has been variable and the evidence of consequences has been mixed. In a study of 12 UK schools, Valentine et al. [41] found that home-school computer uses were poorly linked (e.g., only 10% of students visited their school’s website regularly, and many students and parents were unaware of their schools’ information and computer technology facilities); however, there were modest positive associations between home use of computers for educational purposes and attainment in English and mathematics at some (though not all) school grades. A larger study of 15- to 16-year-olds, conducted in Germany, found no overall relationship between frequency of home use and mathematical attainment [42]. Another large study, using longitudinal panel data collected in the US, found positive associations between computer game play and educational achievement but mixed relationships (varying between genders and demographic groups) for educational uses of computers [30]. Jackson, von Eye, Biocca, Barbatsis, Zhao and Fitzgerald [43], in a study of low-income American adolescents, also reported no link between home internet use (mainly for information-seeking rather than interpersonal communication) and school mathematics attainment, but did find that more time online was associated with higher grade point averages (GPAs) and higher reading scores. Importantly, because of the longitudinal nature of their design, the investigators were able to examine the possibility that superior GPAs predicted subsequent Internet use; there was no evidence of this, supporting the inference that ‘Internet use plays a causal role in academic performance rather than academic performance playing a causal role in Internet use’ (p. 433).

Computers do not bestow educational gains merely by being present or available. The ways in which they are used are diverse, schools’ strategies and teachers’ skills vary, and there are individual differences among students themselves that bear on their readiness to exploit technologies and their success in doing so [43]-[44] [37] [23] [38] [42]. Despite finding no evidence of a general benefit from home use, Wittwer and Sinkbeil [42], for instance, did find that a small group of students who used computers in a deliberated, problem solving way showed positive effects in mathematical attainment. Jackson et al. [43] noted that their evidence of positive impact of Internet use on low-income students may be limited to children in a relatively low performance range (most of their sample were performing below average in school). The authors reasoned that these children may profit from the more intensive engagement in text usage that the Internet fosters, whereas average and more able children may be in less need of this (see also [30]). Broadly compatible results have been reported by Naevdal [45] and Zhu, Chen, Chen and Chern [46]. Naevdal [45] found, among Norwegian adolescents, that time spent working on a personal computer was positively associated with performance in English but, interestingly, this relationship was stronger in children who self-reported as having reading disabilities than in those without reading disabilities. Zhu et al. [46], working with Taiwanese vocational high school students, found that the benefits (in terms of academic performance) of using computers for information seeking were evident in participants with low academic self-efficacy, but not in those who scored high on this variable. Researchers [47]–[48] have also discussed the benefits of computer use for young people with cognitive and/or learning difficulties, such as dyslexia. These young people respond better to online/computer training than person training because computer-based training programmes can be designed to be completely non-judgmental. This can be advantageous to young people who are sensitive about their difficulties; they feel less judged and so are more likely to engage in the training programme.

In sum, while research into the educational benefits of computer use has led to mixed results, several pieces of empirical evidence point to the possibility that, where benefits occur, they are most likely for students with lower abilities; these include those with poorer communication skills [43] and reading difficulties [45]. Individuals with SLI have difficulties in communication and they tend to have poorer than average literacy levels [49]–[51].

Most of the research on new media use and educational achievement available to date has focused on school attainment (ability tests, examinations, GPA). A less investigated, but important, measure of educational outcomes is level of educational progress in the period beyond compulsory education. This is a critical period for determining young people’s access to vocational routes and/or higher education. In general, young people with SLI fare less well than those with TD during this period [19] [52] [7]. In the present study, we were able to collect information on the outcomes at 19 years of young people whose computer uses we examined at around 17 years of age. Researchers have stressed the scarcity of longitudinal research addressing the relationship between home computer use and educational career progress [43] [37] [23] and, to the best of our knowledge, no previous investigations of this topic have examined level of educational progress for young people with language impairment.

We expected that more frequent uses of the home computer for educational purposes should be associated with positive outcomes for participants with SLI. Specifically, we predicted that:

H1: Use of home computers for educational purposes would be associated with positive contemporaneous educational examination achievements in young people with SLI.

H2: For those with SLI, more frequent use of computers for educational purposes at around 17 years would predict more positive educational progress at 19 years.

There was no strong theoretical reason to expect that leisure use would be associated with educational outcomes in this age group; however, we did examine this possibility in order to allow for comparison with the effects predicted for educational uses. For the purposes of this study, we examined concurrent and longitudinal relationships between computer use and educational outcomes in young people with SLI and their TD peers. We were able to demonstrate significant relationships between educational computer uses and level of educational progress two years later in young people with SLI only. However, no such relationships were found for leisure uses of computers and educational outcome for either group.

## Methods

### Ethics Statement

Informed written consent was gained from participants. Ethical approval for the study was obtained from the Senate Committee for the Ethics of Research on Human Beings, The University of Manchester, UK.

### Background of Participants with SLI

The young people with SLI were originally part of a wider longitudinal study, the Manchester Language Study [53]–[55]. This cohort was recruited at 7 years of age from 118 language units attached to mainstream schools in England. Language units are classes that provide intensive language support for children with primary language difficulties (usually) in ordinary schools. They have on average ten children attending, a specialist teacher, a nursery nurse or other type of assistant, and in most cases a half-time speech and language therapist as well [56]. Thus, the staff-student ratio and level of expertise in language units is substantial and placements are offered after a team of trained professionals has assessed referred children (usually prior to school entry) and deemed them to have primary language difficulties, i.e., specific language impairment (SLI). Thus, the participants who volunteered for this study all had primary language difficulties in childhood.

### Participants

Forty-nine young people with SLI (male = 36, 73%) aged between 16 years 2 months and 17 years 10 months (mean age 17;1 years) and 56 typically developing (TD) young people (male = 36, 64%) aged between 16 years 2 months and 17 years 10 months (mean age 16;9 years) volunteered to take part in this two year project. For ease, this will be referred to as ‘around 17 years of age’ throughout. All participants had completed their compulsory education in the UK, had access to a computer at home and spoke British English. The two groups were matched for maternal education level, χ^2^(2, N = 103) = 3.48, p = .176, and household income band, χ^2^(3, N = 103) = 4.97, p = .174.

Table 1 shows the psycholinguistic profiles of both groups. As expected, young people with SLI performed significantly more poorly than TD young people on measures of language. Although both groups of young people had Performance IQ (PIQ) within the normal range, TD young people had significantly higher PIQ than young people with SLI. There appears to be developmental changes in PIQ abilities in individuals with SLI [6]. Young people with SLI tend to have lower PIQ scores than typically developing individuals at older ages, although it is not yet understood why [57].

**Table 1:** Descriptive statistics for young people with SLI and TD peers at around 17 years of age.

	SLI		TD		Comparison	
	N = 49		N = 56			
	M	*SD*	M	*SD*	*t*	*d*
PIQ	93.9	18.2	109.3	9.6	5.78*	1.06
Receptive Language	73.6	18.8	102.4	8.2	10.37*	2.68
Expressive Language	67.1	16.6	104.0	10.2	13.94*	1.99

* p<.001.

### Assessments and Measures

#### PIQ and language assessments

Performance IQ (PIQ) was assessed using the full form of the WASI [58]. The WASI is a battery of four subtests (Vocabulary, Block Design, Similarities, and Matrix Reasoning) and is used to provide a measure of a person’s intellectual ability. It can be used with people aged 6 to 89 years. The Block Design and Matrix Reasoning subtests were used to derive PIQ.

Expressive language, receptive language, and overall core language score were assessed using selected subtests of the Clinical Evaluation of Language Fundamentals – Fourth edition (CELF-4; [59]). The CELF-4 is an individually administered language test designed for 5 to 21-year-olds. Receptive language was assessed using the following subtests: understanding spoken paragraphs, semantic relationships, and the receptive part of Word Classes 2. Expressive language was assessed using the following subtests: Recalling Sentences, Formulated Sentences, and the expressive part of Word Classes 2.

#### Leisure and educational computer use

An interview administered at around 17 years of age contained questions on frequency of home computer use, for both leisure and educational purposes. Two questions on frequency of use queried how often participants used their home computer. One question referred to leisure uses (‘How often do you use your home computer for fun, for example, to play games, browse the web?’) and the other referred to educational uses (‘How often do you use your home computer for school/college work, for example, to search for information, to word process a piece of homework?’). Responses to each question were coded on a four-point scale (1 = less than once a week, 2 = once a week, 3 = two to three times a week, 4 = every day).

#### Examinations at the end of compulsory education

Examination results for standard national tests were available for all participants at the end of compulsory education when they were around 17 years of age. The present study examined General Certificates of Secondary Education (GCSE) examination results in the compulsory subjects of English language, Mathematics and Science. GCSE grades are awarded from A* (highest level) to G (lowest level). Grades were converted into numeric scores using the following point scoring system: 0 = unclassified/failed or not taken, 1 = G, 2 = F, 3 = E, 4 = D, 5 = C, 6 = B, 7 = A, 8 = A*. A composite was calculated for the core subjects by adding up the grade scores for English, Maths and Science (referred to hereafter as GCSE core subject score).

#### Educational and employment status at 19 years

Participants were interviewed concerning their education and employment status at 19 years of age. It was determined whether they were in education, were in full-time or part-time employment or were not in any education, employment or training (NEET). In terms of status at 19 years, 8/49 (16%) participants with SLI and 16/56 (29%) TD participants were in employment and 6/49 (12%) of the SLI group and 3/56 (5%) of the TD group were NEET. Statistical comparisons across groups revealed no significant differences in the proportions in employment (Fisher’s exact *p* = .116) or NEET (Fisher’s exact *p* = .299). However, it needs to be noted that the numbers in each of the cells were small (one cell in the NEET analysis <5), reducing the power to detect differences. The majority of participants, 35/49 (71%) young people with SLI and 37/56 (66%) TD young people were in education.

#### Level of educational progress

If the participants were in education at 19 years of age, the level at which they were studying was determined using national (UK) guidelines (referred to hereafter as level of educational progress). These involve a 7 point scale from Entry level to Level 6. The ordering of the levels represents increasing achievement in education. Thus, Entry level is the most basic level of study and Level 6 is the highest level of achievement in education for the participants’ age group.

Entry level qualifications recognize basic knowledge and skills and the ability to apply learning in everyday situations under direct guidance or supervision. Learning at this level involves building basic knowledge and skills and is not geared towards specific occupations.

Level 1 qualifications (equivalent to General Certificate of Secondary Education (GCSE) grades D-G or Business and Technology Education Council (BTEC) Introductory Diplomas, for example) recognize basic knowledge and skills and the ability to apply learning with guidance or supervision. Learning at this level is about activities which mostly relate to everyday situations and may be linked to job competence.

Level 2 qualifications (equivalent to GCSEs grades A*-C or BTEC Awards, Certificates, and Diplomas at level 2, for example) recognize the ability to gain a good knowledge and understanding of a subject area of work or study, and to perform varied tasks with some guidance or supervision. Learning at this level involves building knowledge and/or skills in relation to an area of work or a subject area and is appropriate for many job roles.

Level 3 qualifications (equivalent to Advanced Level General Certificates of Education (commonly referred to as A-levels) or National Vocational Qualifications (NVQs) at level 3, for example) recognize the ability to gain, and where relevant apply a range of knowledge, skills and understanding. Learning at this level involves obtaining detailed knowledge and skills. It is appropriate for people wishing to go to university, people working independently, or in some areas supervising and training others in their field of work.

Level 4 qualifications (BTEC Professional Diplomas, Certificates and Awards or NVQs at level 4, for example) recognize specialist learning and involve detailed analysis of a high level of information and knowledge in an area of work or study. Learning at this level is appropriate for people working in technical and professional jobs, and/or managing and developing others.

Level 5 (Foundation Degrees or BTEC Professional Diplomas, Certificates and Awards, for example) recognize the ability to increase the depth of knowledge and understanding of an area of work or study to enable the formulation of solutions and responses to complex problems and situations. Learning at this level involves the demonstration of high levels of knowledge, a high level of work expertise in job roles and competence in managing and training others. Qualifications at this level are appropriate for people working as higher grade technicians, professionals or managers.

Level 6 qualifications (Bachelors’ degrees or BTEC Advanced Professional Diplomas, Certificates and Awards, for example) recognize a specialist high level knowledge of an area of work or study to enable the use of an individual’s own ideas and research in response to complex problems and situations. Learning at this level involves the achievement of a high level of professional knowledge and is appropriate for people working as knowledge-based professionals or in professional management positions.

### Procedure

Participants were assessed and interviewed either at home or at school on the above measures, as part of a wider battery at different stages of the longitudinal study. Assessments took place in a quiet room with only the participant and a trained researcher present.

## Results

Descriptive statistics for psycholinguistic profiles, frequency of computer use (educational and leisure), GCSE core subject score at the end of compulsory education and educational progress are presented in Table 2.

**Table 2:** Psycholinguistic profiles, frequency of computer use, GCSE core subject score and level of educational progress for young people with SLI and TD peers continuing in post-compulsory education.

	SLI		TD		Comparison	
	N = 35		N = 37			
	M	*SD*	M	*SD*	*t*	*d*
PIQ	94.6	19.6	110.5	9.7	4.44*	1.03
Receptive language	75.4	19.7	103.4	8.9	7.84*	1.83
Expressive language	67.7	17.1	105.6	10.4	11.46*	2.68
Frequency of educational computer use	2.7	1.1	3.0	0.7	1.56	0.33
Frequency of leisurecomputer use	3.2	0.9	3.4	0.7	0.93	0.25
GCSE core subject score	8.7	6.2	18.3	3.3	8.17*	1.93
Educational progress	2.4	1.9	5.6	1.0	8.92*	2.11

* p<.001.

In terms of level of educational progress, of the young people with SLI, 20% were studying at Entry level, 9% at Level 1, 31% at Level 2, 23% at Level 3 and 17% at level 6. Of the TD young people, 11% were studying at Level 3, 5% at Level 5 and 84% at Level 6. The level of educational placement was coded as follows: 0 = Entry level to 6 = Level 6. The mean level of young people with SLI (M = 2.4, SD = 1.9) was significantly below that of TD young people (M = 5.6, SD = 1.0), *t*(70) = 8.92, *p*<.001, *d* = 2.11.

The correlations among frequency of computer use, expressive language, receptive language, PIQ, GCSE core subject score at around 17 years and level of educational progress at 19 years are presented in Table 3. For both groups, patterns of correlations involving GCSE core subject scores revealed no significant associations with frequency of computer use (neither educational nor leisure). In contrast, significant correlations were found between GCSE core subject scores and expressive language (EL) and receptive language (RL) as well as PIQ. Note that expressive and receptive language were strongly correlated in the SLI group (*r* = .85) and moderately correlated in the TD groups (*r* = .56).

**Table 3:** Correlations between frequency of computer use and key variables for young people with SLI and TD peers continuing in post-compulsory education.

	Freq of educational computer use	Freq of leisure computer use	Expressive language	Receptivelanguage	PIQ	GCSE core subject score
Freq of leisurecomputer use	.17 [.14]	.				
Expressive language	.29 [.12]	.15 [.47**]	.			
Receptive language	.26 [.24]	.32 [.18]	.85** [.56**]	.		
PIQ	.36* [.26]	.21 [.19]	.50** [.34*]	.59** [.54**]	.	
GCSE core subject score	.30 [-.03]	.13 [.12]	.66** [.44**]	.69** [.36*]	.50** [.44**]	.
Level of educational progress	.49** [.18]	.20 [.07]	.57** [.05]	.62** [.06]	.57** [.40*]	.81** [.49**]

First number denotes SLI (*n* = 35) and number in square bracket denotes TD (*n* = 37).

* p<.05, **p<.01.

Patterns of correlations involving level of educational progress at age 19 were different for adolescents with SLI and TD peers. The pattern of correlations also indicated different relationships for different types of computer uses. Thus, regression analyses were carried out separately for each group for each type of use (educational versus leisure).

### What Predicts Examination Outcome?

Hierarchical regression analyses were conducted to examine the contribution of PIQ, expressive language, receptive language and frequency of computer use (leisure use and educational use separately) to compulsory examination outcome. In each model, PIQ was entered as a first step to control for the effects of nonverbal ability.

For both young people with SLI and TD young people, frequency of leisure computer use did not make a significant contribution to GCSE core subject score over and above PIQ.

In terms of frequency of educational computer use, for young people with SLI, the model predicting GCSE core subject score was significant at step 1, *F*(1,32) = 10.54, p<.01, *f* ^2^ = .33 (moderate effect size), and at step 2, *F*(4,29) = 7.56, *p*<.001, *f* ^2^ = 1.04 (large effect size). After accounting for PIQ, there was a trend for receptive language to make a significant contribution to GCSE core subject score (*p* = .065).

For TD young people, the model predicting GCSE core subject score was significant at step 1, *F*(1,35) = 8.30, *p*<.01, *f* ^2^ = .24 (moderate effect size), and at step 2, *F*(4,32) = 3.64, *p*<.05, *f* ^2^ = .45 (large effect size). After accounting for PIQ, expressive language made a significant contribution to GCSE core subject score (*p* = .025), explaining 6.9% of unique variance.

In sum, as shown in Table 4, PIQ contributed to the prediction of examination outcomes. Once this factor was controlled for, a borderline contribution was made by receptive language in the SLI group. For the TD group, expressive language made a significant contribution to explaining variance in examination outcomes. The same pattern of results was found when the full sample of young people was used (including those individuals who did not continue in education post-compulsory schooling, i.e., 49 SLI and 57 TD).

**Table 4:** Hierarchical regression analysis predicting GCSE core subject score separately for young people with SLI and TD peers continuing in post-compulsory education.

Variable	Inc. *R* ^2^	*F*-change	β	*t* value	part corr^2^
SLI					
Step 1	.24	14.78***			
PIQ			.49	3.85***	.24
Step 2	.33	10.79***			
PIQ			.10	.81	.01
Expressive language			.29	1.57	.03
Receptive language			.38	1.89^a^	.04
Frequency of educational computer use			.13	1.22	.02
TD					
Step 1	.20	13.53**			
PIQ			.45	3.68**	.20
Step 2	.35	3.91*			
PIQ			.26	1.96^b^	.05
Expressive language			.32	2.32*	.07
Receptive language			.06	.41	.00
Frequency of educational computer use			.17	1.44	.03

* p<.05; **p<.01; ***p<.001.

a p = .065, ^b^p = .055.

Note: SLI total *R* ^2^ = .51, Adj *R* ^2^ = .44; TD total *R* ^2^ = .31, Adj *R* ^2^ = .23.

### Does Frequency of Computer Use Predict Subsequent Educational Progress?

Further regression analyses were conducted to examine the contribution of frequency of computer use at around age 17 years to level of educational progress at 19 years. Separate regression models were carried out for leisure computer uses and educational computer uses. In each model, GCSE core subject score was entered as a first step to control for educational qualification level. PIQ, expressive language, receptive language and frequency of computer use were entered as predictors in the second step.

For both young people with SLI and TD young people, frequency of leisure computer use did not make a significant contribution to level of educational progress over and above GCSE core subject score.

In terms of frequency of educational computer use, for young people with SLI, the model predicting level of educational progress was significant at step 1, *F*(1,32) = 62.15, p<.001, *f* ^2^ = 1.94 (large effect size), and at step 2, *F*(5,28) = 16.15, p<.001, *f* ^2^ = 2.89 (large effect size). After accounting for GCSE core subject score, only frequency of educational computer use made a significant contribution to level of educational progress (*p* = .030), explaining 4.8% of unique variance.

For TD young people, the model was significant at step 1, *F*(1,35) = 10.64, p<.01, *f* ^2^ = 0.30 (moderate effect size), and at step 2, *F*(5,31) = 3.81, p<.01, *f* ^2^ = 0.61 (large effect size). However, after accounting for GCSE core subject score, it was found that PIQ, expressive language, receptive language and frequency of educational computer use did not make an additional significant contribution to level of educational progress.

In sum, as seen in Table 5, as would be expected, GCSE core subject score contributed to the prediction of subsequent educational progress in both groups. Once this factor was controlled for, in the SLI group frequency of educational use of computers made a significant, unique contribution to the young person’s educational progress. In contrast, in the TD group, no significant additional contribution was made by educational uses of computers.

**Table 5:** Hierarchical regression analysis predicting level of educational progress separately for young people with SLI and TD peers continuing in post-compulsory education.

Variable	Inc. *R* ^2^	*F*-change	β	*t* value	part corr^2^
SLI					
Step 1	.66	62.15***			
GCSE core subject score			.81	7.88***	.66
Step 2	.08	2.24^a^			
GCSE core subject score			.67	4.90***	.22
PIQ			.14	1.11	.01
Expressive language			−.08	−.39	.00
Receptive language			.07	.34	.00
Frequency of educational computer use			.24	2.29*	.05
TD					
Step 1	.23	10.64**			
GCSE core subject score			.44	3.26**	.23
Step 2	.15	1.84			
GCSE core subject score			.52	3.02**	.18
PIQ			.31	1.71^b^	.06
Expressive language			−.18	−.98	.02
Receptive language			−.23	−1.21	.03
Frequency of educational computer use			.19	1.24	.03

* p<.05;

** p<.01;

*** p<.001.

a p = .090,

b p = .098.

Note: SLI total *R* ^2^ = .74, Adj *R* ^2^ = .70; TD total *R* ^2^ = .38, Adj *R* ^2^ = .28.

## Discussion

The relationship between home based computer use and educational attainment is controversial and previous results have been mixed. Where positive relationships have been reported, they have tended to obtain in groups of adolescents with poorer educational records and/or developmental impairments. We predicted that frequency of educational computer use would be positively associated with examination achievements and level of educational progress in a sample of young people with SLI. This was not supported for examination achievements but was supported for educational progress two years later. We had not expected frequency of leisure uses to be a significant predictor of educational outcomes for either group, and there was no evidence to indicate such a relationship. As expected, educational benefits appear to reflect specifically educational uses, rather than entertainment and interpersonal activities.

### Frequency of Leisure Use and Educational Progress

Frequency of leisure use was not related to exam performance at the end of compulsory education or to subsequent progress. Within the age range sampled here, the link between ‘fun’ uses of computers and ‘serious’ educational work does not appear to be strong.

These results, however, should not be taken to indicate that leisure use of home computers is irrelevant to educational attainment in young people with SLI or TD. It is possible that any impact due to leisure uses occurs earlier. For example, fun uses of computers at home may ease children’s route into computer use at school [23]. It is worth noting that there was no indication of a negative relationship. That is, there is no reason to suppose, on the basis of the present non-significant correlational findings, that leisure uses of computers are contrary to young people’s educational prospects.

It is also possible that any benefits of leisure use are found in the social domain, rather than in educational outcomes. For example, adolescents who use computers as part of interpersonal communications, shared games, or other joint activities are participating in interactions with peers and may practise social skills or learn about others’ characteristics and perspectives in these contexts. Social relations with peers tend to be problematic for children and adolescents with SLI [60]–[61] and the fact that they engage in leisure uses of home computers as much as do adolescents with TD may mean that they find this a more manageable medium for social relations than face-to-face interactions. A limitation of this study is that measures in the social domain were not included. This is an area that can be addressed in future research.

The present study examined frequency of use but not quality of use. Future research is also needed to examine the quantity and quality of leisure uses among young people at different age points. A limitation of the present research is that we did not distinguish among different types of leisure use, and it may be that different activities have different consequences.

### Frequency of Educational Use and Educational Progress

Educational use of computers at home was not significantly associated with examination results for young people with SLI or young people with TD. In contrast, PIQ and both expressive and receptive language skills were significantly correlated with GCSE core subject scores. For both groups, regression analyses confirmed that, once PIQ was controlled for, only language skills made a borderline (SLI) or significant (TD) contribution to explaining variance in examination results. This pattern of results indicates that, regardless of whether they have SLI or not, young people with higher intellectual abilities, who are more likely to do well in examinations at school, are also likely to have better language skills. The significant correlation observed between expressive and receptive language in both groups suggests both sets of skills are likely to be implicated.

Educational computer use at home does not appear to be strongly linked to examination outcomes. However, the positive, albeit non-significant, correlation between educational computer use and GCSE core subject score for the SLI group (*r* = .30) suggests this is an interesting area for future research. The effects of educational computer use may be more subtle than we had anticipated; a larger sample size may be required to observe them and to gain a more complete understanding of their potential contribution.

Nevertheless, educational uses of computers at around age 17 was strongly correlated with level of educational progress in young people with SLI some two years later. Regression analyses showed that educational uses did contribute to the prediction of progress, once national examination scores at around age 17 were controlled for. Thus, it appears that engagement in this study medium is facilitative of progress during an important transition period, as the young people move beyond compulsory schooling. Why should this be the case?

As pointed out by others [23] [42], there has been a tendency in the literature to assume that computers, especially educational computing, must be ‘good’ for young people but relatively little attention has been paid to the ways in which any benefits occur. We suggest that several interrelated factors are likely to be implicated in explaining the present findings for participants with SLI. Educational computer use can either be a marker for other factors which directly affect educational progress or it can be an influential factor itself. We discuss these two different types of interpretation in turn.

First, much is likely to depend on the user’s motivations and commitment. As argued by Wittwer and Senkbeil [42], home uses of computers for educational purposes are likely to draw on the user’s problem solving orientation. Kirkorian and Anderson [44] have argued that mental effort is a crucial determinant of the effects of young people’s engagements with new media. Durkin et al. [12] found that adolescents with SLI tended to find work with educational applications challenging. Not all students are disposed to invest in problem solving and difficult tasks, and some adolescents with SLI do avoid or minimize educational uses of home computers. The present findings are consistent with the interpretation that those who persist in the face of these challenges profit because they are spending time purposefully, investing mental effort to determine how to use the applications for their needs.

Second, it is likely that readiness to use home computers for educational purposes is correlated with readiness to undertake homework and academically-linked activity *per se*. More frequent use of computers and the Internet is likely to require more reading [43], and the practice of literacy skills is likely to support educational activity. We did not have independent measures of extent of homework or reading in this study, and this should be taken into account in future research.

Third, positive outcomes in educational progress are not simply a matter of hard work paying off. Investment in developing specific skills that have transferable potential may also be important. Thus, frequency of using computers for leisure purposes (which could also involve effort and at least some problem solving) does not appear to predict educational progress in this age group, but frequency of using them for educational purposes does.

Fourth, success in computer uses tends to promote computer-related self-efficacy [13] [62] and self-efficacy influences subsequent progress in education [63]. It is likely that, as students improve their skills in the domain, positive feedback and increasing sense of mastery nourish their self-confidence as computer users (and possibly beyond).

With regard to educational computer use being an influential factor itself, we consider a more speculative proposal, i.e. that using computers when undertaking study is beneficial for the language and literacy performance of young people with SLI, and that any gains in these respects are in turn advantageous to educational progress. It must be stressed that this hypothesis has not been tested directly in the present study, because we did not have language or literacy measures at age 19. However, the positive association between frequency of educational computer use and level of educational progress in the SLI group only is consistent with the assumption that some characteristics of this particular group are enhanced in the course of the activity. Computer-based study can be self-paced, often provides instant feedback, and furnishes frequent examples of written language in the context of goal-directed tasks (finding information, completing set tasks, preparing reports). Hetzroni and Schreiber [64] reported better literacy performance (including spelling and textual organization) in young adolescents introduced to use of a word processing package, in comparison to their performance in traditional handwriting tasks. Durkin, Conti-Ramsden and Walker [65] found that adolescents with SLI reported higher language-related motivations for using computer mediated communication (relaxed spelling in emails, being able to type instead of having to talk, and control of time needed to write and read) than did adolescents with TD. Future research could address the possibility that those who devote time and effort to these kinds of activities experience improvements, or greater confidence in, their linguistic performance.

In sum, we suggest that home uses of computers for educational purposes are predictive of educational progress during this phase of adolescence for those with SLI in part because they reflect the young person’s readiness to face challenging learning tasks and to persist in the face of difficulties. In addition, working in this way is likely to yield specific gains due to developing skills and confidence with the equipment that will be increasingly utilized in educational and occupational settings, and possibly some advantageous practice in linguistic and literacy performance.

### Adolescents with Typical Development

We did not preclude the possibility that positive relationships would also obtain in adolescents with TD. Indeed, several of the arguments in the preceding section concerning the benefits of educational uses of computers might also be expected to hold for TD students. Frequency of educational uses of home computers, however, did not contribute to the prediction of examination results or subsequent educational progress in these participants.

It may be that there are positive consequences of using computers among typical children but that these occur earlier in this group and have plateaued by the age range tested here. For example, as argued at the outset, computer use depends partly on linguistic skills; the relevant abilities may be consolidated earlier in TD [66] and so phases where there could be facilitation in either direction may have already passed. Another possibility is that use of this mode of study has little material impact on typical adolescents’ school performance: there are various ways to undertake school work and homework, and typical children may find the routes that suit them. As noted in the Introduction, evidence concerning the consequences of educational computing is equivocal but, where benefits have been identified, they have tended to be among students with lower abilities and exceptional characteristics [43], [45]. Our results are consistent with this pattern. This is not to suppose that benefits for those with TD are not possible, but they were not evident in the present context and sample.

### Implications and Conclusions

Some 7% of children begin their school lives with SLI. Although they are within the normal intellectual range, their linguistic difficulties mean that these young people face continuous challenges throughout their education. The present findings indicate that using home computers for educational purposes may make a positive contribution to their educational progress during an important transition phase in adolescence. This is striking because the condition itself appears to be stable in adolescence [6] and because available evidence indicates that young people with SLI receive little additional support in terms of how to use educational programmes and software [12]. Thus, the findings suggest scope for targeted interventions to exploit what appear to be propitious uses of new technology. We need also to bear in mind the substantial subset of adolescents with SLI who tend not to use home computers for educational purposes [12]. Further research in this area with larger samples of adolescents with SLI is warranted. In addition, both basic and applied research are needed to understand what is beneficial and how it can be developed to support optimal outcomes for young people with SLI.
